# Thoracic Endovascular Aortic Repair for an Aortoesophageal Fistula Caused by Esophageal Cancer: A Case Report and Literature Review

**DOI:** 10.7759/cureus.64243

**Published:** 2024-07-10

**Authors:** Pham Thao Vy Le, Hong Thoai Nguyen, Vinh Sieu Lam, Hieu D Nguyen, Thi Anh Thao Chu

**Affiliations:** 1 Cardiovascular Research Laboratories, Methodist Hospital, Merrillville, USA; 2 Internal Medicine, Ascension Saint Joseph Hospital, Chicago, USA; 3 Internal Medicine, Yavapai Regional Medical Center, Prescott, USA

**Keywords:** gastrointestinal bleeding, chemotherapy, thoracic endovascular aortic repair, esophageal cancer, aortoesophageal fistula

## Abstract

Aortoesophageal fistula (AEF) is an uncommon complication of esophageal cancer and can be extremely fatal if left untreated. Compared to open repair, thoracic endovascular aortic repair (TEVAR), a less invasive technique, is the initial recommended treatment in cases of hemorrhagic shock secondary to AEF, as this procedure showed a favorable outcome in controlling the overt bleeding. Here, we present a case of a patient with a history of stage IV esophageal cancer being treated with chemotherapy and an esophageal stent due to a previous tracheoesophageal fistula who presented to the emergency room due to severe gastroesophageal bleeding and hemorrhagic shock. A CT angiography of the chest revealed an AEF. The patient was subsequently resuscitated and treated with TEVAR. After the procedure, the hemorrhage was managed, and the patient was discharged with palliative radiation therapy. However, after one month, the patient had a major gastrointestinal hemorrhage, which caused her death. This example indicates the necessity of early detection and surgical intervention in AEF patients with unstable hemodynamics who have underlying unresectable esophageal cancer and chemotherapy. TEVAR should be conducted as soon as possible before the open surgery to achieve the best outcome for patients.

## Introduction

Aortoesophageal fistula (AEF) is an uncommon but life-threatening disorder caused by an abnormal connection between the aorta (or aortoiliac tree) and the esophagus. Esophageal cancer is the third leading cause of AEF [1], with thoracic aortic aneurysm and congenital abnormalities accounting for most causes of primary AEF. Secondary AEF (SAEF) most frequently involves the presence of extraneous objects, such as aortic stent grafts [2]. Risk factors for the formation of AEF include malignancy, radiation therapy [3], and direct injury from a previous esophageal stent [4]. Thoracic endovascular aortic repair (TEVAR) is the recommended initial treatment for patients with severe hemorrhagic shock caused by AEF [5]. However, the long term is associated with higher rates of recurrent infection and bleeding, particularly for the sole use of TEVAR without intestinal repair [6]. Definitive repair of the esophagus was achieved with open graft explantation, resection, and repair. Open surgery is associated with elevated rates of both mortality and morbidity in these cases [2,3]. Most patients with AEF die before receiving a diagnosis or undergoing surgical treatment. In this study, we provide a case of AEF in a patient with unresectable esophageal cancer, which was effectively managed by TEVAR.

## Case presentation

**Figure 1:**
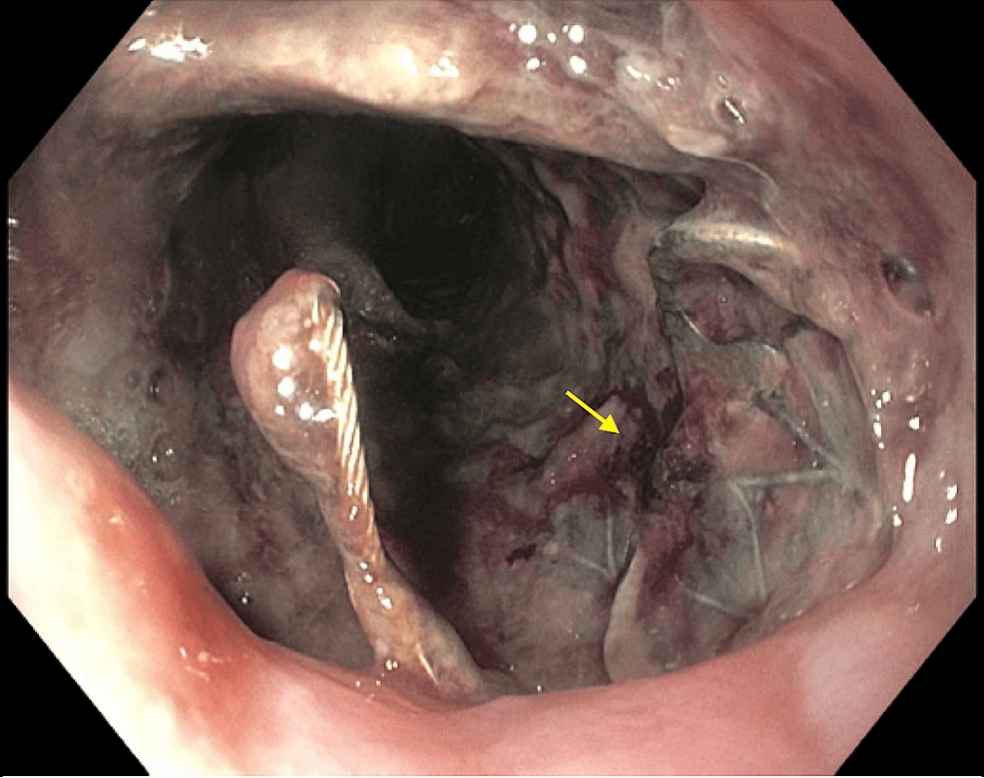
Endoscopy showing a large, fungating mass with stigmata of recent bleeding (yellow arrow) in the entire esophagus, 20 cm from the incisors

**Figure 2:**
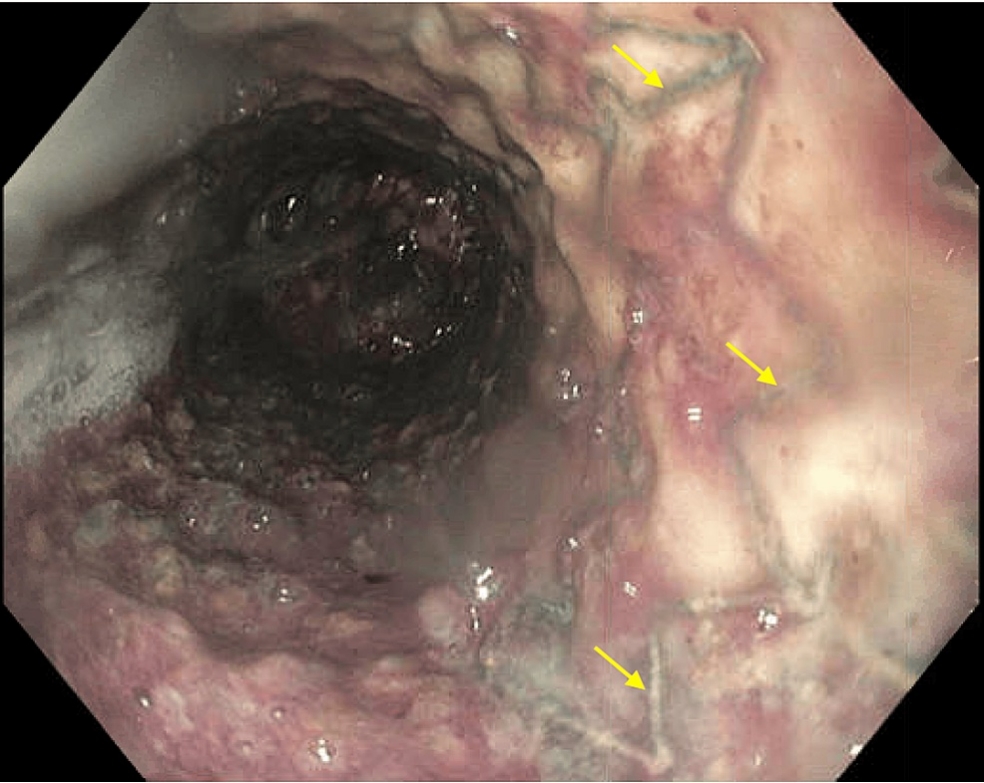
An esophageal stent was identified (yellow arrows), spanning the entire esophagus from 20 cm to 35 cm. The tumor was non-obstructive and circled the esophagus

A 60-year-old female patient with a history of stage IV esophageal squamous cell carcinoma was receiving chemotherapy. She also had an esophagotracheal fistula and pulmonary abscess and underwent esophageal stent placement. Recently, she developed thrombosis in the right brachial and left carotid veins and was treated with enoxaparin. The patient was admitted to the emergency room due to hematemesis and hypotension. She presented with three episodes of hematemesis and melena over the past two days. She also reported non-radiating chest pain and palpitations. Upon arrival at the emergency room, her blood pressure was 78/53 mmHg, her pulse was 93 beats per minute, and her hemoglobin level was critically low at 4.3 g/dl (12.0-15.3 g/dl). She received a transfusion of four units of red blood cells, a normal saline infusion, a norepinephrine drip, and pantoprazole 40 mg IV every 12 hours. Enoxaparin was discontinued, and she received one unit of fresh frozen plasma. Gastroenterology was consulted for an urgent endoscopy, which revealed a large, circumferential, non-obstructive mass with diffuse oozing and recent bleeding in the entire esophagus, located 20 cm from the incisors (Figure 1). The tumor was non-obstructive and encircled the area. There was widespread oozing from multiple parts of the tumor that could not be resolved through endoscopic treatment. An esophageal stent was present throughout the esophagus and was able to pass through (Figure 2). With the current treatment, the patient's hemodynamics improved, and her hemoglobin level increased to 8.1 g/dl (12.0-15.3 g/dl) (Table 1). Subsequently, she was transferred to the medical floor for further care.

**Table 1:** The patient's hemoglobin on admission and after treatment MCV: mean corpuscular volume; MCH: mean corpuscular hemoglobin; MCHC: mean corpuscular hemoglobin concentration

Component	Reference range	On admission	After transfusing four units of red blood cell
Hemoglobin (g/dl)	12.0-15.3	4.3	8.1
MCV (fL)	80.0-100.0	95.4	89.8
MCH (pg)	26.0-34.0	31.3	31.0
MCHC (%)	32.5-35.8	32.8	34.5

The following day, she experienced persistent nausea and continued to have occasional episodes of vomiting blood without black, tarry stools. Although her hemodynamics remained stable, her blood pressure was low, and her hemoglobin levels began to decrease. After withholding enoxaparin for a day, she developed swelling in both upper arms, despite an ultrasound examination showing no signs of a blood clot in the deep veins. She received a fifth unit of red blood cells and was then transferred back to the intensive care unit.

**Figure 3:**
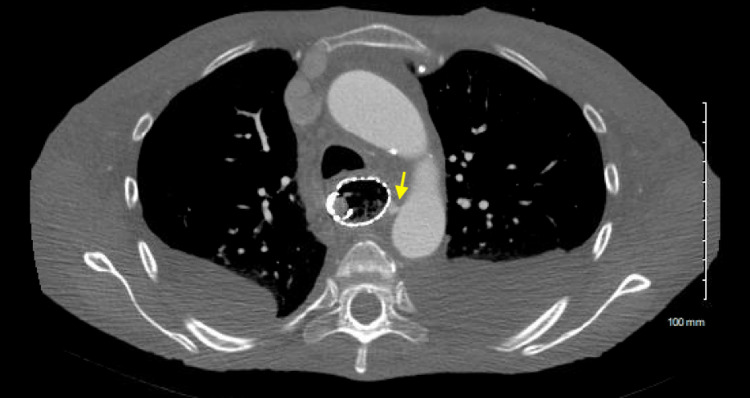
CT angiogram showing punctate aortoenteric fistula extending from the proximal descending aorta to the proximal esophagus (yellow arrow). This fistula was covered by the existing esophageal stent, without overt extravasation into the esophageal lumen

In the intensive care unit, the patient experienced continuous blood coughing and bloody bowel movements, causing hemodynamic instability. She required multiple units of red blood cells and fresh frozen plasma to maintain her blood pressure. Due to ongoing gastrointestinal bleeding and the need for a massive transfusion, the interventional radiology team was consulted to perform arterial embolization following a CT angiogram. The CT angiogram showed an esophageal mass with an aortoenteric fistula originating from the proximal descending aorta to the stented proximal esophagus without significant bleeding into the esophageal lumen (Figure 3). Vascular surgery performed endovascular exclusion of the AEF using a thoracic aortic stent graft. The patient also received palliative radiation therapy to control bleeding from the esophageal tumor during her hospital stay and continued the treatment after discharge.

Regrettably, one month later, the patient experienced recurrent bleeding and became hemodynamically unstable. A follow-up CT angiogram revealed a reoccurrence of the AEF. Vascular surgery recommended urgent TEVAR, but the patient opted not to undergo the procedure as it was considered a temporary solution. Subsequently, the decision was made to transition the patient to palliative care, and she passed away the following day.

## Discussion

AEF is an uncommon but life-threatening disorder caused by an abnormal connection between the aorta (or aortoiliac tree) and the esophagus. The primary symptom of AEF is profuse bleeding in the upper gastrointestinal system. Causes of primary AEF include thoracic aortic aneurysm, which accounts for the majority of cases at 54.2%, followed by malignancy, congenital anomalies, and stomach reflux [1]. Esophageal cancer is the third leading cause of AEF, as evidenced by a survey of 500 cases, which highlights the correlation between AEF and malignant tumors in the thorax [1]. Carcinoma infiltrating the aorta can lead to tumor necrosis and subsequently cause AEF. SAEF most commonly involves the presence of extraneous objects, such as aortic stent grafts [2] and foreign bodies. SAEF may also occur with other procedures involving the aorta, such as endovascular aneurysm repair, and other endovascular interventions, such as the utilization of bare metal aortic stents [7]. In this case, the patient with a history of stage IV esophageal cancer being treated with chemotherapy and an esophageal stent due to a tracheoesophageal fistula presents with typical symptoms of AEF, such as massive bleeding that leads to hemorrhagic shock. Esophageal cancer invasion, chemotherapy [3], and direct injury from a previous esophageal stent [4] all contribute to the formation of the fistula in this patient.

The occurrence of AEF in cancer patients who are receiving chemoradiation therapy can be attributed to radiation, either independently or in conjunction with chemotherapy [8]. Definitive chemoradiotherapy (dCRT) is the preferred therapy for inoperable esophageal squamous cell carcinoma that has not metastasized. The occurrence rate of AEF when treated with this method is approximately 10-12%, particularly if patients have a total circumferential lesion and a C-reactive protein (CRP) level of 1.00 mg/dl or higher [9]. Evidence of radiation-induced injury has been provided by the numerous cases of a patient with extraesophageal cancer who had been irradiated prior to the manifestation of an AEF [10]. Desquamation of endothelial cells, formation of fibrous tissue, thrombus formation, and blockage are all observed in small arteries and arterioles. These arteries, the vasa vasorum in particular, are especially susceptible to the harmful impacts of radiation, leading to the rupture of the primary blood vessels [6,11]. In this case report, esophageal stenosis being treated with an esophageal stent due to tracheoesophageal fistula greatly enhances the probability of AEF formation in patients receiving chemotherapy for locally advanced, inoperable thoracic esophageal squamous cell carcinoma, as seen in this patient [12].

Vascular surgery is the primary and essential treatment for AEF and should be performed as soon as feasible. TEVAR is a minimally invasive technique that involves inserting a covered stent into the aorta. TEVAR is the recommended initial treatment for patients with severe hemorrhagic shock caused by AEF [5]. TEVAR was initially developed in 1996 to address the management of a fistula between the aorta and a neighboring organ [13]. TEVAR has demonstrated efficacy in the treatment of thoracic aortic aneurysms and other conditions affecting the thoracic aorta [14]. TEVAR offers expedited and safer surgical intervention, serving as a bridge in unstable patients, followed by definitive open repair, to promptly manage bleeding, restore circulation, and prevent overt rapid exsanguination [15]. In some cases, endovascular methods can be used for the palliative treatment of patients with high surgical risk. TEVAR has a very high rate of technical success (87.3%) and a more favorable 30-day mortality rate (19.7%), improving early outcomes compared with immediate open surgery [2,16]. In the context of esophageal cancer, TEVAR can increase the average survival period by four months in cases of AEF [17]. 

While TEVAR is a less invasive approach, it still has its own set of restrictions when it comes to treating AEF with well-documented issues related to vascular and infectious consequences. The long term is associated with higher rates of recurrent infection and bleeding, particularly for the sole use of TEVAR without intestinal repair, at 13.8% and 15.2%, respectively [6]. In TEVAR, patients do not receive additional esophageal repair, and the stent graft remains in contact with a contaminated environment and continues the infection. Therefore, infectious outcomes such as mediastinitis and persistent sepsis are notable, and extended antibiotic therapy is strongly correlated with increased mortality. Aside from infectious issues, the insertion of aortic endovascular grafts is linked to device-related complications such as component segregation, stent-graft bowing, and displacement over a period. In addition, for individuals with an AEF that has already ruptured, it is recommended to provide open thoracic surgery instead of TEVAR. In this case report, the patient with AEF and hemorrhagic shock underwent TEVAR. The patient's bleeding was rapidly controlled after inserting the stent graft, and she was discharged with prolonged antibiotics and a follow-up for palliative radiation.

Definitive repair of the esophagus was achieved with open graft explantation, resection, and repair. This procedure was successfully performed within one month of presentation in 11% of patients [6]. In open repair procedures, the mortality rate was high, ranging from 45.4% to 55% [2,3]. The major complications after an open repair include surgical site infection (3%), venous thromboembolism (1.7-4.2%) [14], and postoperative bleeding (approximately 10% after urgent or emergency surgery) [18]. Late complications include the development of a hernia, anastomotic aneurysm or pseudoaneurysm, and aortic graft infection. 

If left untreated, AEF is almost always lethal [16]. Despite advancements in resuscitation techniques and surgical intervention, AEF remains correlated with significant rates of morbidity and mortality. An analysis of 118 cases of AEF revealed an overall mortality rate of 86% and a surgical mortality rate of 36% [19]. Most patients die before receiving a diagnosis or undergoing surgical treatment. The outcomes are affected by the timing of the diagnosis, the patient's medical condition, the severity of the infection, and the exact location of the afflicted aorta. Early diagnosis and aggressive surgical intervention are needed to obtain the best outcome for patients. In this case report, the patient underwent TEVAR, which successfully controlled the bleeding. After one month of discharge, the patient presented to the emergency room due to massive bleeding and was subsequently diagnosed with a recurrent AEF, which caused her death. Her complicated medical condition with underlying stage IV esophageal cancer and immunocompromised state due to chemotherapy and prior esophageal stenosis being treated with an esophageal stent, along with TEVAR as the sole treatment for AEF, all contributed to the challenges in diagnosis and poor outcome in this patient.

## Conclusions

This case report describes the clinical presentation and effective treatment of TEVAR in a patient with a rare case of AEF and esophageal cancer. TEVAR is a highly efficient intervention that should be promptly conducted to control overt bleeding from AEF. It serves as a temporary method for subsequent definitive aortic vascular repair, especially in patients who are not suitable candidates for open procedures or curative therapy. However, the high rates of recurrent bleeding and persistent infection after TEVAR, particularly in the context of an underlying unresectable malignancy and immunocompromised state secondary to chemoradiation therapy, all contribute to the complexity and poor prognosis in these patients. Prolonged antibiotics should be maintained to prevent infectious complications after TEVAR, which is strongly associated with improved survival rates in these patients. Definitive repair of the esophagus should follow TEVAR with open graft explantation, resection, and repair to achieve the best outcome for patients.
